# Lactic Acid Bacteria in Vinegar Fermentation: Diversity, Functionality and Health Benefits

**DOI:** 10.3390/foods14040698

**Published:** 2025-02-18

**Authors:** Elahesadat Hosseini, Zenebe Tadesse Tsegay, Slim Smaoui, Theodoros Varzakas

**Affiliations:** 1 National Nutrition and Food Technology Research Institute, Faculty of Nutrition Sciences and Food Technology, Shahid Beheshti University of Medical Sciences, Tehran 1981619573, Iran; hosseinielahe14@gmail.com; 2 Department of Chemical Engineering, Payame Noor University, Tehran 1659639884, Iran; 3 Department of Food Science and Post-Harvest Technology, College of Dryland Agriculture and Natural Resources, Mekelle University, Mekelle P.O. Box 231, Ethiopia; ztlovewith73@gmail.com; 4 Laboratory of Microbial and Enzymatic Biotechnologies and Biomolecules, Center of Biotechnology of Sfax (CBS), University of Sfax, Road of Sidi Mansour Km 6, P.O. Box 1177, Sfax 3018, Tunisia; slim.smaoui@cbs.rnrt.tn; 5 Department of Food Science and Technology, University of the Peloponnese, Antikalamos, 24100 Kalamata, Greece

**Keywords:** lactic acid bacteria, fermentation, bioactive compounds, functional food, diversity, vinegar production

## Abstract

Vinegar, frequently distilled by solid fermentation or liquid processes, was generated through the synergistic effect of a microbial community in open or semi-open environments. Based on the studied raw materials, researchers distributed the vinegar into three classes: grain, fruit and animal, with lactic acid bacteria (LAB) playing a pivotal role in their fermentation and contributing significantly to their functional and sensory qualities. Typically, the natural maturation of fresh vinegar necessitates a long period and vast space, engendering a reduced efficiency. To accelerate the vinegar aging process, some physical methods, viz. micro-oxygenation, ozone, ultrasound, microwave, gamma rays, infrared, electric fields and high pressure, have been developed. Produced or enriched by LAB, key bioactive vinegar components are organic acids, phenolic compounds, melanoidins, and tetramethylpyrazine. These active compounds have antibacterial, antioxidant, anti-inflammatory functions; aid in the regulation of liver protection metabolism and glucose control; and have blood pressure, anti-tumor, anti-fatigue and metabolic regulatory effects. The review explores advancements in vinegar production, including modernized fermentation processes and optimized aging techniques, which enhance these beneficial compounds and ensure product consistency and safety. By examining the LAB variety strains and the bioactive profiles of different vinegar types, this study highlights vinegar’s value beyond a culinary product, as a potential therapeutic agent in human nutrition and health. The findings underscore vinegar’s relevance not only in dietary and preventive healthcare but also as a potential functional food ingredient. Further research is needed to explore the mechanisms of action through which LAB contribute to the development of several new healthy vinegars.

## 1. Introduction

LAB can be employed as a probiotic, offering major health benefits [1], and has lately been linked with dairy and non-dairy products due to the high demand for gluten-free and lactose-free products [2]. Vinegar constitutes one of the earliest culinary creations, representing an ancient fermented food with a long history that dates back to 2000 BCE [3]. Its sedative and curative properties make its application as a remedy unique, with such use reported in the Bible [4]. It can be employed as a preservative, a flavor enhancer, but also as a cleaning agent for surfaces and utensils [5]. The natural fermentation of vinegar involves the presence of LAB [6]. LAB species utilize plants, cereals, fruits, whey, and honey as physical support or as carbon and energy sources, which are usually introduced at the initial stage of vinegar fermentation [7]. Even at lower concentrations, LAB can be constant and/or can dominate vinegar fermentation [8,9].

For vinegar manufacture, raw material contribute substantially to its final features. As an illustration, an evaluation of 83 types of aroma profile was undertaken by Natera et al. [10]. Predicated on their raw materials, the chemometric investigation was based on isopentanoic acid, 2,3-butanediol, 2-phenylethanol, 3-hydroxy-2-butanone and 3-methylbutanol, with the balsamic and the apple vinegars assembled in one area and the alcohol, honey and cider vinegars clustered together. Ozturk et al. [11] analyzed the aroma profiles of Turkish vinegar made by industrial and traditional methods. These authors reported that isoamyl acetate and ethyl acetate were found in a vinegar made from grapes and that octanoic acid was produced in pomegranate vinegar. On the other hand, Chinese vinegar prepared from different cereal products viz. barley, rice, wheat, corn and sorghum—popularly known as Qu—employed *Monascus*, *Rhizopus*, *Aspergillus*, LAB and *Saccharomyces* (yeast) as the main microorganisms. According to the raw materials and the used microorganism, Qu has been presented in a multitude of forms. The *Maiqu* was found to derive from wheat in which *Aspergillus* spp. was employed as the main microorganism. *Xiaoqu* and *Daqu* from rice, wheat, peas, and barley. For vinegar fermentation, the dominant microorganisms were *Rhizopus* spp. and *Mucor* spp. for *Xiaoqu* and *Daqu*, respectively [12]. Additionally, the initial status (e.g., canned, fresh or frozen) of the fruit can disturb the content of bioactive compounds, such as polyphenols and vitamins. Consequently, it is probable that the chemical composition of the original fruit will affect the final vinegar characteristics. For instance, Al-Dalali et al. [12] investigated the physicochemical, microbiological and bioactive properties of fruit vinegars manufactured from various raw materials. These authors reported that all of the studied parameters have an impact on the fruit vinegars; in addition, high levels of total phenolic content (TPC) and total flavonoid content (TFC) could engender high biological activity.

Concerning the volatile compound composition in the vinegars found from diverse fruits, Coelho et al. [13] outlined the impact of *Acetobacter* metabolism on the aroma profile. These authors stated that fruit vinegars displayed high contents of minor volatiles linked to varietal aroma. For orange, cherry, banana and mango vinegars, major volatile profiles were presented by monoterpenic alcohols and by C_13_-norisoprenoids, benzaldehyde, esters and furaneol, respectively. All these data support the relevance of the raw material to the qualities of the end product.

Vinegar harnesses antibacterial properties and fosters tissue repair and this is ascribed to the extracellular structure synthesized by *Acetobacter* species [14]. It might also protect against oxidative stress-related injuries [15], also promoting anti-inflammatory and anti-cancer activities. Excretion of calcium works for acetate for the prevention of kidney stone formation [1], which can also reduce glucose blood levels by helping its conversion to glycogen [16]. In light of these pharmacological effects, vinegar is ranked as a latent drug candidate in the treatment of several clinical conditions—mainly those connected to hyperlipidaemia, diabetes and obesity—and can decrease plasma triglyceride and cholesterol contents. It has an antidiabetic effect which is due to the action of acetic acid, as reported by the previous authors, and a hypotensive effect has been reported for red wine vinegar by inhibition of the renin–angiotensin system in rats [17]. Acetic acid was found to suppress body fat accumulation [18]. In this sense, 0.57 mM of acetic acid could reduce the activity of plasma aldosterone and plasma renin. In addition, in an in vivo model, 0.2% of acetic acid diminished xylulose5-phosphate accumulation in the liver and phosphofructokinase-1 activity in skeletal muscle [19]. Vinegar’s consumption benefits are directly influenced by the bioactive compounds produced by LAB, such as those isolated from tangerine vinegar, which include organic acids, polyphenols, and melanoidins. These compounds are known for their antioxidant, anti-inflammatory, and metabolic-regulating properties, which are linked to the health benefits of vinegar [20]. On the other hand, some of the organic acids and bioactive components of vinegar (e.g., acetic acid, gallic acid, catechin, epicatechin, chlorogenic acid, caffeic acid, p-coumaric acid, and ferulic acid) [14], could play a crucial role in various physiological processes, such as the absorption of minerals, by lowering the gastrointestinal pH [21]. Several contemporary studies have been achieved to confirm the requested effects of vinegar, employed in folk medicine against an extensive variety of ailments and infections like warts head lice, wounds, ear infections insect bites, and persistent cough [22].

This review aims to compile the overall microbial diversity of LAB in vinegar and identify its functional and bioactive compounds along with its health benefits in terms of its use as a postbiotic supplement in human nutrition. We have searched important and relevant databases, such as Scopus, Science Direct, and Researchgate, with the combination of keywords such as lactic acid bacteria, fermentation, vinegar, health benefits, diversity, and functionality.

## 2. Diversity and the Functional Role of LAB in Vinegar Fermentation

Vinegar fermentation involves a remarkable diversity of LAB, surpassing that of acetic acid bacteria (AAB). LAB encompass six genera and 26 species, with major genera including *Lactobacillus*, *Pediococcus*, *Leuconostoc*, and others. These species play distinct roles at various stages of fermentation, such as early fermentation, flavor development, and health-promoting compound production [23]. LAB are extensively present in various vinegar types including apple [24], rice [25,26], cereal [27,28], Shanxi-aged [8], Zhejiang rosy [6], Zhenjiang aromatic vinegar [29,30], Qishan [31], Tianjin Duliu [32], Sichuan sun vinegar [33,34,35], rose vinegar [36], malt vinegar [37], and persimmon vinegar [38], highlighting their significant role throughout the fermentation process [6]. The dominant genus, *Lactobacillus*, constitutes over 70% of the LAB population and includes key species such as *L*. *plantarum*, *L. casei*, *L. acetotolerans*, and *L. fermentum* [32]. Following closely, the *Pediococcus* genus predominantly features *P*. *acidilactici* and *P*. *pentosaceus.* Additionally, species such as *Weissella confusa*, *Lactococcus lactis*, *Leuconostoc mesenteroides*, and *Oenococcus* contribute to the complex microbial ecosystem during vinegar production [31]. The presence and dominance of different LAB genera vary across fermentation stages and vinegar types. For example, *Lactobacillus* is prevalent in most types except for rice and cereal vinegar [25,27]. Additionally, *Pediococcus* and *Lactococcus* are dominant in Shanxi-aged vinegar [8] and Daqu starter [39], while *Weissella* is common in Tianjin Duliu and Qishan vinegar [31,32]. *Leuconostoc* thrives in Qishan vinegar, and *Oenococcus* is associated with apple vinegar fermentation [40].

LAB typically dominate the early and middle stages of vinegar fermentation, often in symbiosis with yeasts such as *Saccharomyces cerevisiae*. Their population generally ranges between 10^2^ and 10^4^ CFU/g, while the total microbial population in vegetables and fruits varies between 10^5^ and 10^7^ CFU/g. Despite these population differences, LAB play a crucial role in fermentation due to their metabolic activity, contributing essential amino acids and vitamins that shape the fermentation process [41], quality and the end product’s fermentation process. These synergies are competitive and symbiotic, engaging multifaceted dynamics that are determined by many factors, such as temperature, nutrient availability, osmotic stress, and acetic acid concentration [42]. In the initial fermentation stage, yeasts are the principal microorganisms generating byproducts such as acetaldehyde, organic acids, EtOH and glycerol [43]. These byproducts can influence AAB development, the liberation of free amino acids and the autolysis of yeast cells [44]. This symbiosis is beneficial for vinegar manufacture, generating a promising environment for the growing of yeasts and AAB and improving the fermentation efficiency [45]. LAB strains which produce lactic acid and additional organic compounds can contribute to the vinegar’s acidity and flavor [42]. LAB can also synthesize bacteriocins that prevent the progress of other microorganisms, such as AAB [46]. Even if yeasts deliver essential nutrients (e.g., amino acids and vitamins) for AAB and LAB, these latter can participate for O_2_ and nutrients, possibly affecting microbial population dynamics and fermentation progression [42]. The interaction of yeasts/AAB/abiotic variables such as O_2_ availability, temperature, and nutrient levels are indispensable for preserving an effective fermentation process and creating high-quality vinegar. Hence, in order to optimize the fermentation efficiency and achieve a desired sensory profile, it is crucial to manage and control these interactions and environmental variables. Regarding O_2_, its absence decreases the AAB fermentative capacity [47]; nevertheless, high levels could damage yeast by oxidative stress and form reactive oxygen species, which harm cellular components [47]. Osmotic stress from high sugar concentrations also affects yeast ethanol production [48,49]. The temperature also influences the microbial interactions in vinegar production. Low temperatures delay the fermentation [50], while extreme temperatures can endorse unwanted microorganisms’ development, disturbing vinegar quality [42].

Throughout fermentation, for both yeasts and AAB, the nutrient availability is decisive. Yeasts synthesize essential nutrients like amino acids and vitamins that assist the growth of AAB [44]. Conversely, AAB may provide competition for these nutrients, to a large extent following alcoholic fermentation, once nutrient levels become exhausted.

In turn, LAB, particularly species such as *Lactobacillus plantarum*, *Lactobacillus acetotolerans*, and *Pediococcus acidilactici*, play a significant role in the early stages of fermentation. These species are responsible for lactic acid production, which lowers the pH of the fermentation medium. This process creates an optimal environment for the formation of polyphenols and melanoidins, compounds known for their antioxidant and anti-inflammatory properties [51]. For instance, during the initial 10–30 days of Zhejiang rosy vinegar fermentation, *Lactobacillus* is predominant; however, *Acetobacter* becomes more abundant during the later stages [6]. LAB abundance tends to decline as the fermentation process progresses into acetic acid fermentation (AAF) due to their low tolerance for acidic conditions [52]. However, species such as *L. acetotolerans* and *L. fermentum* persist, demonstrating their adaptability to low pH environments. Concurrently, the population of acetic acid bacteria (AAB), including *Acetobacter* and *Gluconacetobacter* species, increases as they thrive in the acidic conditions required for vinegar production. The composition and abundance of AAB can vary depending on the type of vinegar and its geographical origin, influenced by raw materials, fermentation methods, and environmental factors. Studies have shown that traditional vinegars from different regions harbor distinct microbial communities, leading to unique flavor profiles and fermentation kinetics. During the initial stages of fermentation, yeasts and LAB dominate, producing ethanol and lactic acid, which serve as substrates for AAB. In the intermediate stages, LAB populations decline while AAB populations expand, facilitating the conversion of ethanol into acetic acid. Despite this, species such as *L. acetotolerans* and *L. fermentum* persist, demonstrating their adaptability to low pH environments [8,32].

Beyond their role in microbial dynamics, LAB play a critical part in improving the sensory characteristics and safety of vinegar [9]. They produce lactic acid, which lowers pH and imparts a fresh and mild sourness that balances the strong acidity of acetic acid [30,32]. LAB also generate volatile compounds like esters and aldehydes, contributing to fruity, creamy, and buttery flavors in vinegar, which in turn enhance the sensory quality of the final product. Furthermore, melanoidins produced during fermentation are known for their antioxidant properties, which contribute to the health-promoting effects of vinegar, such as oxidative stress reduction [52,53]. For example; the key microorganisms responsible for the flavor of Zhenjiang aromatic vinegar (ZAV) include *Lactobacillus acetotolerans*, *Lactobacillus plantarum*, *Lactobacillus reuteri*, *Lactobacillus fermentum*, and *Acetobacter pasteurianus*, as reported by Ye et al. [29]. Additionally, bacteriocins produced by LAB inhibit the growth of spoilage and pathogenic microorganisms [6]. Their metabolic adaptability allows LAB to survive and function in harsh conditions by altering cell membrane composition and employing proton pumps to maintain intracellular pH balance [30]. Furthermore, LAB such as *L. fermentum* are known to inhibit the formation of harmful advanced glycation end-products (AGEs), enhancing both the safety and sensory appeal of the final product [54]. Overall, the diversity and functional applications of LAB in vinegar fermentation underscore their indispensable role in producing high-quality vinegar with desirable sensory and microbial properties.

## 3. Vinegar Functional Compounds

Different types of vinegar contain bioactive compounds such as organic acids, polyphenols, melanoidins, and tetramethylpyrazine. These compounds have been shown to offer a range of health advantages, including antibacterial and antioxidant properties. Regular vinegar consumption may contribute to weight management, improved blood pressure and glucose control, and better vascular health [14,16,55].

### 3.1. Organic Acids

Vinegar’s acidity stems from a combination of organic acids. These acids can be categorized into volatile acids, such as acetic, formic, propionic, butyric, and quinic acids, and nonvolatile ones, including lactic, malic, pyroglutamic, citric, and succinic acids [16,56,57]. The origins of these organic acids can be traced back to both the fermentation process and the raw materials used. Among these, acetic acid is the predominant organic acid in vinegar, formed during acetic acid fermentation, while lactic acid, a byproduct of alcohol fermentation, is present in much lower concentrations [32,56,58,59]. Additionally, fruits contribute a range of organic acids, such as malic, citric, tartaric, lactic, succinic, and γ-aminobutyric (GABA) acids, to the overall composition of fruit vinegar [56,60,61]. Organic acids in vinegars are mostly produced by microorganisms throughout the fermentation phase [62] and are classified as volatiles or nonvolatile acids. The main organic acid is the acetic acid which is produced by AAB through the fermentation stage [38]. Lactic acid, principally a nonvolatile organic acid in vinegars, is mostly generated in the alcoholic fermentation stage. However, tartaric acid, malic acid, citric acid and propionic acid are found during the progression of the fermentation [16]. In addition, the fermentation milieu also impacts the organic acid levels. As an illustration, during the fermentation process, steaming, heating, and fermenting tools could reduce the volatile organic acid concentrations, increasing the levels of nonvolatile organic acids and thus the ratios of nonvolatile/volatile organic acids [16].

Acetic acid in vinegar influences weight loss through the following mechanisms: (i) lessening the lipids synthesis, (ii) accumulation of oxygenolysis and excretion of lipids, (iii) augmentation of postprandial satiety, and (iv) raising energy consumption by increasing myoglobin levels and upregulating the expression of genes related to the synthesis of fatty acids [16].

The organic acids in the different types of vinegar are shown in Table 1. Among these, acetic acid reigns supreme, constituting a substantial portion (30–50%) of the total organic acid content in various vinegar types [63]. While acetic acid is the predominant volatile organic acid, lactic acid holds the title of the most abundant nonvolatile organic acid [61,64,65,66]. Other organic acids, such as formic, citric, malic, and succinic acids, have also been identified in vinegars, particularly balsamic vinegar, through advanced analytical techniques like nuclear magnetic resonance (NMR) [67]. Acetic acid, with its potent aroma and flavor, is a significant contributor to vinegar’s sensory profile. However, the presence of lactic, tartaric, malic, and succinic acids acts as a counterbalance, resulting in a milder overall taste [63]. Beyond their organoleptic properties, organic acids in vinegar offer nutritional and functional benefits [63]. Compounds such as malic, citric, succinic, and lactic acids can be metabolized through the tricarboxylic acid cycle, a fundamental pathway for energy production from carbohydrates, lipids, and amino acids. Moreover, these acids, especially acetic acid, exhibit antimicrobial properties. Acetic acid, in particular, has demonstrated potent efficacy against harmful bacteria such as *E. coli* O157:H7 [55]. Its antibacterial action is further enhanced when combined with citric acid. In addition to antimicrobial effects, acetic acid has garnered attention for its potential health benefits. Animal studies have indicated that acetic acid can contribute to lipid management by reducing cholesterol and triglyceride levels through mechanisms involving liver lipogenesis inhibition and increased bile acid excretion [68]. Furthermore, it has been shown to influence body fat accumulation by upregulating genes associated with fatty acid oxidation in the liver. The research of Shen et al. [69] provides compelling evidence for the potential therapeutic benefits of acetic acid and vinegar in inflammatory bowel disease (IBD), specifically colitis. Their study has demonstrated that both acetic acid (0.3% *w*/*v*) and vinegar (5% *v*/*v*) are effective in alleviating the symptoms of colitis induced by dextran sulfate sodium (DSS) in mice.

### 3.2. Phenolic Compounds

The phenolic compounds that enrich vinegars primarily originate from raw materials used in their production. Research has consistently identified a variety of phenolic acids within grain-based vinegars, including those derived from sorghum, bran, barley, pea, and rice bran. These phenolic acids encompass a diverse range of compounds such as gallic, ferulic, syringic, vanillic, protocatechuic, caffeic, chlorogenic, p-coumaric, p-hydroxybenzoic, sinapic, and salicylic acids [66,79,80]. Fruit vinegars, derived from fruits such as apples, grapes, pomegranates, blueberries, and black wolfberry, are abundant sources of phenolic acids. These compounds include catechin, syringic acid, gallic acid, chlorogenic acid, epicatechin, caffeic acid, ferulic acid, rutin, protocatechuic acid, and p-coumaric acid [61,81,82,83]. In each type of vinegar, the type and levels of polyphenols are different. For instance, during the fermentation steps, grain vinegars mostly comprise phenolic acids as ferulic acid, sinapic acid and protocatechuic acid [56], and the amounts of gallic acid and catechins are small. Conversely, the two phenolic compounds are higher in fruit vinegars [16]. The fermentation process could influence the types and contents of polyphenols. For instance, acetic acid fermentation reduces the polyphenol content of vinegars [56], and the wooden bottles employed in the aging process could impact the kinds and levels of polyphenols present in fruit vinegars [16].

Phenolic acids are renowned for their antioxidant properties. They effectively neutralize harmful free radicals such as hydroxyl radicals and superoxide anions through electron transfer, thereby preventing chain reactions. Additionally, these compounds can chelate with metal ions, inhibiting oxidation processes [84]. A detailed overview of the phenolic acid profiles in different vinegar types can be found in Table 2. A comparative analysis of phenolic compounds in various vinegars revealed significant disparities in their composition. For instance, Zhenjiang aromatic vinegar exhibited the highest concentration of gallic acid (555.3 ± 2.32 mg/L), while red wine vinegar was characterized by the highest abundance of caftaric acid (176.61 ± 0.24 mg/L) [65,85]. These findings underscore the notion that the specific phenolic profile of vinegar is contingent upon both the raw materials used and the manufacturing processes involved. A study by Budak et al. [86] revealed that traditionally produced grape wine vinegar contains higher levels of chlorogenic and syringic acids compared with its industrially produced counterpart. Conversely, industrial grape wine vinegar exhibited a greater concentration of catechin. Regarding antioxidant capacity, as measured by oxygen radical absorbance capacity (ORAC) and Trolox equivalent antioxidant capacity (TEAC) values, traditional grape wine vinegar outperformed the industrial variety. Additionally, both types of grape wine vinegar demonstrated superior antioxidant properties when compared with apple cider vinegar [86,87,88]. Furthermore, studies by Bertelli et al. [89] and Xie et al. [90] have indicated a positive correlation between aging time and the overall polyphenol content in vinegars. This suggests that the aging process significantly influences the phenolic composition of vinegar products. Research consistently demonstrates the potent antioxidant properties of vinegar phenolic compounds, contributing to reduced oxidative stress, improved lipid metabolism, blood pressure regulation, cardiovascular health, liver protection, and anti-aging effects. These benefits are largely attributed to their ability to neutralize free radicals, enhance endogenous antioxidant enzyme activity (such as superoxide dismutase and catalase), and regulate redox-sensitive signaling pathways, including Nrf2 and NF-κB, which modulate oxidative stress responses. A strong correlation exists between phenolic content and antioxidant capacity in vinegars, highlighting their role in promoting cellular defense mechanisms against oxidative damage [56,90,91]. Comparative studies have shown that traditional vinegar production methods yield products with higher antioxidant activity than those produced industrially [82,92]. Analysis of traditional balsamic vinegars revealed that both melanoidins and polyphenols significantly contribute to their antioxidant properties [93,94,95]. Vinegar studies commonly identify gallic, caffeic, and catechin acids as prevalent phenolic compounds. However, flavonoid content in vinegars remains relatively low. A notable exception is pomegranate vinegar, which Kharchoufi et al. [96] found to contain 17 phenolic compounds—a significantly higher number than in other vinegars—as determined by UPLC-MS analysis. Protocatechuic acid, as a predominant phenolic acid in pomegranate vinegar, was the most abundant compound, followed by gallic acid. It is one of the key polyphenols contributing to the bioactivity of vinegar.

Cell and animal studies have shown that polyphenols in both grain and fruit vinegars possess potent antioxidant properties [89,97,98]. These compounds can effectively reduce oxidative stress and protect hepatocytes from damage by activating the Nrf2 signaling pathway [90,99]. Furthermore, vinegar polyphenols have been shown to lower blood lipid levels, regulate blood glucose, prevent blood clots, and exhibit anti-tumor effects [86,91,100,101]. Like blood glucose metabolism, acetic acid in vinegar could reduce the synthesis of lipids and increase the decomposition and excretion of lipids by the AMPK activating pathway [102]. During the transformation of acetic acid into acetyl-CoA, the AMPK activation pathway resulted in a decrease in the cholesterol concentration, triglycerides, and LDL by downregulating the expression of the srebp-1 gene. Moreover, triggered AMPK constrains the expression of a series of genes linked to fatty acid synthesis across the carbohydrate phosphorylation response element binding protein (ChREBP), which decreases the synthesis of fatty acids [102]. Additionally, acetic acid diminishes the blood lipid levels of rats by stimulating the oxygenolysis of fatty acids and the secretion of bile [16].

These findings collectively highlight the significant health benefits of vinegar consumption, including improved cardiovascular health, liver protection, and overall well-being.

**Table 2:** Phenolic compounds in various types of vinegars.

Vinegar Types	Phenolic Compounds	References
Traditional balsamic vinegar	Furan-2-carboxylic, 5 hydroxyfuran-2-carboxylic,4-hydroxybenzoic, vanillic, protocatecuic, syringic, isoferulic, pcoumaric, gallic, ferulic and caffeic acids	[103]
Balsamic vinegar	Protocatechuic, gallic, p-coumaric, syringic, caffeic, ferulic, vanillic, salicylic, homovanillic, hydroxytyrosol, gentisic, p-carboxyphenol, protocatechuic, sinapinic and p-hydroxybenzoic acids and phenol, catechin, aesculetin, epicatechin, vanillin, coniferyl alcohol, 4-methylcatechol, syringaldehyde, isopropiovanillone, scopoletin, aceto-/isoacetovanillone, isopropiosiringone, acetosyringone, isoacetosiringone, syringol, coniferylaldehyde, sinapinaldehyde, tryptophol, o-vanillina, methyl vanillate, (m + p)-cresol, 4-ethylcatechol, ocresol, vanillyl ethyl ether, guaiacol, 4-methylsyringol, 4-vinylphenol, ethyl vanillate, 3,4- xylenol, 4-vinylguaiacol, ellagic acid, 4-ethylphenol, 4-methylguaiacol, 4-ethylguaiacol, 4-allylsyringol, eugenol and isoeugenol	[103,104]
Grape vinegar	Gallic, chlorogenic, caffeic, syringic, and ferulic acids and catechin and epicatechin	[82]
Sherry vinegar	Gallic acid, Ellagic acid, protocatechuic, caffeoylquinic acid, protocatechualdehyde, tyrosol, p-OH-benzoic acid, catechin, p-OH-benzaldehyde, siringic, vanillin, caftaric, cis-p-coutaric, trans-p-coutaric, fertaric, caffeic, cis-p-coumaric, trans-p-coumaric, ferulic acids and quercetin 3-o-galactoside, quercetin 3-oglucuronide, kaempferol 3-o-galactoside, pelargonidine 3-o-galactoside, pelargonidine 3-o-robinobioside and aromadendrin 7-o-glucoside	[105,106]
Apple vinegar	Gallic, vanillic, chlorogenic, caffeic, p-coumaric, trans-ferulic, 4-pcoumaroylquinic, pcoumaroylquinic, p hydroxybenzoic, and protocatechuic acids, and (−)-epicatechin gallate and phloridzin	[65,107]
Apple cider vinegar	Gallic acid, catechin, epicatechin, chlorogenic acid, caffeic acid, and p-coumaric acid	[86]
Persimmon vinegar	Gallic, chlorogenic, caffeic, p-coumaric, trans-ferulic, Hydroxycinnamic, acids, (−)-epicatechin gallate, gallocatechin gallate, procyanidin A2, rutin epigallocatechin phloridzin, catechin hydrate, and flavanols	[65,99]
Red wine vinegar	Gallic acid, protocatechuic acid, caffeic acid, vanillic acid, catechin, epicatechin, caftaric acid, syringic acid, ellagic acid, p-coumaric acid, ferulic acid and chlorogenic acid	[82,85]
Shanxi aged vinegar	Protocatechuic, p-hydroxybenzoic, salicylic, dihydrosinapic, p-coumaric, sinapic, dihydroferulic and ferulic acids	[108]
Pomegranate vinegar	Gallic acid, punicalagin, catechin, vanillic acid, syringic acid, galloylglucoside, protocatechuic acid, ethyl gallate, ellagic acid, chlorogenic acid, caffeic acid, p-coumaric acid, ferulic acid, ferulic acid hexoside, tyrosol and trans-p-Coumaric derivates	[96]
Zhenjiang aromatic vinegar	Gallic, vanillic, chlorogenic, p-coumaric and trans-ferulic acids, epicatechin and catechin hydrate	[65]

### 3.3. Melanoidins and Tetramethylpyrazine

Melanoidins are complex brown molecules formed during the Maillard reaction between sugars and proteins [109,110], and are abundant in vinegars due to thermal processing and aging. Thermal treatment in grain vinegar production breaks down complex compounds into simpler sugars and amino acids, which then react to create melanoidins [111]. As reported by Liu et al. [112], in vinegars, melanoidins are principally made in steaming and baking (grain vinegars), and the aging process (fruit and grain vinegars). In addition to the reaction of amino acids and sugars, phenolic compounds in vinegars can also polymerize with melanoidins, becoming a part of their skeleton, which enhances the antioxidant potential [16].

Aging focuses on these compounds and incorporates phenolic compounds from wood in fruit vinegar [105,113]. These high-molecular-weight melanoidins (10–100 kD) [114] are potent antioxidants, effectively binding metals [115] and gaining additional antioxidant power from incorporated phenolic acids [116]. Beyond their impact on flavor [109], melanoidins in vinegar like Zhenjiang aromatic and traditional balsamic exhibit strong antioxidant properties [117]. These compounds significantly contribute to the vinegar’s overall antioxidant capacity, as measured by 2,2′-azino-bis (3-ethylbenzothiazoline-6-sulfonic (ABTS) and ferric-reducing antioxidant power (FRAP) assays [117], and protect the liver from oxidative damage [118]. Additionally, melanoidins demonstrate antibacterial activity against common pathogens [119].

Tetramethylpyrazine (TMP), a compound produced via Maillard reactions and microbial fermentation, is a key component influencing the characteristics of vinegar. Its concentration fluctuates throughout the vinegar production process. For instance, TMP levels in Shanxi-aged vinegar increase during acetic acid fermentation after a low initial concentration [120]. In tartary buckwheat vinegar, TMP content rises initially but declines after three days of thermal processing [121]. Conversely, TMP accumulates over time in Zhenjiang aromatic vinegar [59]. A broader study of 137 vinegars revealed higher TMP levels in solid-state fermented vinegars compared with liquid-fermented ones [99,122,123], suggesting that thermal and aging processes are crucial for TMP formation. Beyond its impact on flavor, TMP offers potential health benefits. It has been linked to inhibiting platelet aggregation, vasodilation, lipid reduction, and antioxidant activity [124,125].

### 3.4. Other Bioactive Compounds

Vinegar’s diverse applications stem from unique production methods and specialized raw materials. For instance, Monascus-aged vinegar contains lovastatin, a byproduct of microbial fermentation, which has been linked to reduced blood lipid and pressure levels [126]. Baoning vinegar, a renowned Chinese product, undergoes a meticulous process involving Daqu preparation, fermentation, boiling, and filtration. The addition of medicinal herbs like *Amomum villosum*, *Elettaria cardamomum*, *Eucommia ulmoides*, and *Angelica sinensis* to Daqu endows the vinegar with therapeutic properties, including spleen fortification, stomach nourishment, and overall health enhancement.

Intriguingly, Cao et al. [127] discovered fluorescent nanoparticles (FNs) in mature Chinese vinegar. These approximately 1.40 ± 0.40 nm spherical particles are hypothesized to originate from the breakdown and restructuring of polysaccharides and proteins during the vinegar-making process. The interaction of these FNs with dopamine suggests potential health implications, warranting further investigation into vinegar’s multifaceted benefits.

## 4. Functional Quality and Safety Improvements of Vinegar

The functional quality and safety of vinegars will have an impact on consumers’ health. The bioactive components present in vinegar, such as organic acids, melanoidins, polyphenols, and tetramethylpyrazine, can impact antioxidant activity, protect the liver, control blood pressure and glucose, regulate lipid metabolism, control anti-fatigue and anti-tumor activity, promote digestion, stimulate the appetite, exert antidiabetic effects, and show antimicrobial properties [56,128]. Bouazza et al. [129] investigated lipid metabolism and liver damage in orally dosed high-fat-fed rats with fruit vinegars made from pomegranate [*Punica granatum* L. (Punicaceae)], prickly pear [*Opuntia ficus-indica* (*L.*) Mill. (Cactaceae)], and apple [*Malus domestica* Borkh. (Rosaceae)]. They reported that these fruit vinegars regulate lipid metabolism and decrease liver damage in high-fat-fed rats.

Vinegar produced by spontaneous fermentation is vulnerable to consistent quality and complexity of microbiota. Therefore, the fermentation process should be monitored, controlled, and optimized to enhance the safety, flavor, and health benefits of the final product, i.e., vinegar. To avoid these challenges, modern biological technology and vinegar fermentation have applied the following strategies [130]: (1) Application of statistical techniques (partial least squares regression, two-way partial least squares regression, etc.) and metagenomics techniques used to identify the genetic potential of fermenting microbiota by detecting genes associated with metabolic pathways. (2) Isolation of strains showing high-temperature tolerance, acid tolerance, and tolerance under pressure conditions using modern biological technology. (3) Regulation of the key driving forces of the fermentation process using metatranscriptomic, metaproteomic, and metabolomics analysis methods. (4) Utilization of bottom-up approaches, including classical microbiology and single-cell technology, along with top-down metagenomics, to enhance the qualitative and quantitative analysis of microbiota throughout the fermentation process. This approach enables precise microbiota modeling, contributing to the standardization and modernization of vinegar production. Statistical multivariate data investigation have confirmed their strong ability to offer a high possibility of the complete characterization and identity authentication of vinegars. Liu et al. [131] concurrently identified the tartaric, acetic and formic acids, and pH of different vinegar fruits (peach, apple and lemon) by NIR spectroscopy linked to LS-SVW. PLS models were established with different preprocessing methods, and the performance of the LS-SVM models was related with three different kinds of inputs, including wavelet transform (WT), latent variables (LV), and effective wavelength by regression coefficients, x-loading weights, modeling power, and ICA. To predict organic acids, findings revealed that LS-SVM models were superior to PLS models. Regarding pH in different fruit vinegars, EW-LS-SVM models were better than LV-LS-SVM models. Liu and He [132] used the same method to predict pH and soluble solids content (SSC) to categorize the varieties of four fruit vinegars. Throughout fermentation, results indicated that it was possible to classify fruit vinegars according to their pH and SSC. By an NIR spectroscopy technique coupled with the LS-SVM algorithm, sugar content in fruit vinegar was predicted by Wang et al. [133]. These authors confirmed that PCA and LS-SVM models could be practically applied to the accurate and fast determination of sugar contents in fruit vinegar. Artificial neural networks (ANNs) have been linked to UV–vis spectroscopy to distinguish between vinegars produced by diverse raw materials [134] and vinegars mixed with spirit vinegar or acetic acid [135]. These techniques showed statistical discrimination efficiency.

Metagenomics is employed to assess the metabolic potential, classification, and diversification of microbial members in the diverse biosynthesis pathways of vinegar microbiota. For instance, Amplicon metagenomics employing approaches such as Illumina sequencing has been implemented to classify and assess AAB metagenomes in several varieties of vinegar [136]. To understand the potential metabolic functions encrypted by the genomes of the communal members, shotgun metagenomics was utilized for examining the cereal vinegar microbiota [137]. These authors used an effective composition and phylogeny-based algorithm to categorize shotgun metagenomics in order to understand the flavor-producing microbiota and functional groups in Chinese cereal vinegar. These authors detected seven amino acids, three organic acids, and twenty volatiles as leading vinegar metabolites. A metabolic network for substrate analysis and principal flavor generation in vinegar microbiota was created, and microbial distribution discrepancy in different metabolic pathways was registered [137].

This technique revealed the metabolic system liable for flavor. Zhu et al. [52] studied the bacterial dynamic progression and flavor development in three batches of Shanxi aged vinegar (SAV) using high-throughput sequencing and metabolomics approaches. On the basis of its bacterial community succession, acetic acid fermentation stage (AAF) can be separated into three stages (early, (days 0–4); medium (days 5–21); and later (days 22–26)). *Rhizobium*, *Lactococcus Pantoea* and *Pediococcus* contributed significantly in the early stage. However, *Lactobacillus* was predominant in the medium stage and *Komagataeibacter Kroppenstedtia* and *Acetobacter*, were the major bacteria in the later stage. Throughout the AAF, 42 volatile compounds and 7 organic acids were obtained. By using Spearman correlation analysis, a significant link between bacterial community/favor metabolites during the AAF of the SAV was displayed.

On another study conducted by Li et al. [120], *Lactobacillus Escherichia*, and *Klebsiella Acetobacter* were noticed in the AAF using the g denaturing gradient gel electrophoresis (DGGE) technique. By metatranscriptomic approach, Huang et al. [138] concluded that the dominant species in a starter vinegar are *Lactobacillus acetotolerans*, *L*. *helveticus*, *Acetilactobacillus jinshanensis* and *A*. *pasteurianus*. During acetic acid fermentation, a link was established between these microorganisms.

**Figure 1:**
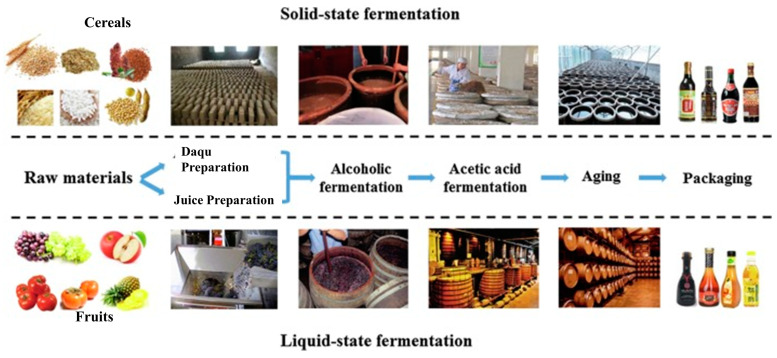
Raw materials and fermentation methods of vinegar production (reproduced from Xia et al. [56] with permission from *Journal of Functional Foods*, copyright 2020).

Generally, the methods employed to produce vinegar are classified as the slow method (Orleans method) and the rapid methods (submerged and generator methods) [128]. Traditional vinegar production passes through five common processes: steaming the raw material, two-stage fermentation, fumigation, vinegar pouring, and aging. Figure 1 shows the sources of materials, fermentation methods, and other common processes during vinegar production.

### 4.1. Vinegar Quality Improvement

The quality of vinegars is dependent on finding appropriate additives, selecting perfect raw materials, the mechanism of controlling environmental factors, and the mechanisms of microbial biotransformation [130].

Molds, yeasts, and bacteria degrade proteins. Carbohydrates and fats present in the fermentation substrate affect the final vinegar quality by secreting enzymes such as amylase, glucoamylase, lipase, and proteases during fermentation [130].

Currently, special vinegars are produced by employing different acetification methods and the addition of extracts. In particular, Bertan et al. [139] produced specialty vinegars from pineapple processing residues by enriching them with phenolic contents and antioxidant potential from Red-Jambo *Syzygium malaccense* leaf extract. The enriched specialty vinegars were found to contain polyphenols (443.6–337.3 mg GAE/L). Moreover, the acetification method that was applied reduced the saturation index and was able to intensify the color of the final vinegar.

The maturation of young vinegar for an extended time helps to improve its flavor. The following latest advanced vinegar flavor maturation regulation technologies are currently in development [140]: (1) Microbial fortification or multi starter fermentation, in which fermentation byproducts, like total acids, esters, and aroma precursors, facilitate vinegar flavor maturation. (2) The optimization of key production processes, such as oxygen flow, and fermentation temperature and the adjusting of raw material compositions enhance its flavor by generating alcohols, organic acids, polyphenols, and esters. (3) Applying novel physical processing, like ultrasonication, ultra-high pressure, and microwave treatment, promotes the conversion of alcohols into acids and esters in vinegar, which reduce flavor maturation time by over six months. For instance, Wang et al. [63] investigated the application of slim ultrasonic treatment to accelerate Zhenjiang vinegar maturation. They reported that many mature vinegar quality indicators, such as a reduction of total amino acid from 1082.259 mg/100 mL to 871.045 mg/100 mL, enhanced volatile components (total esters, aldehydes and heterocyclic), and an increase of total non-volatile organic acids from 202.59 mg/10 mL to 233.87 mg/10 mL were achieved at an optimum ultrasonic power of 50 W/100 mL, a time of 75 min and an addition of 0.75% (*v*/*v*) ethanol for aging vinegar.

**Figure 2:**
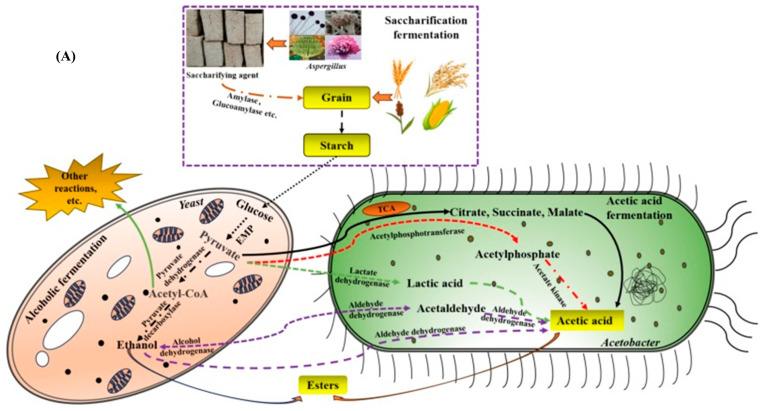

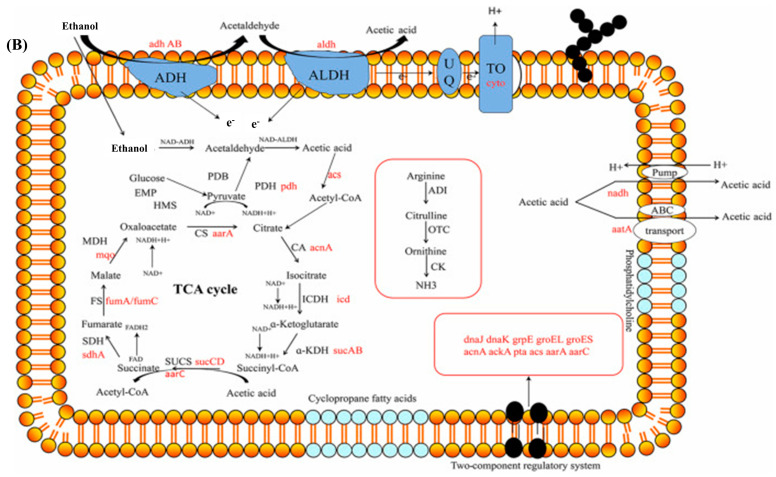
Mechanisms of vinegar fermentation and their Impact on final product quality. (**A**) Fermentation process of traditional Chinese vinegar production (reproduced from Zhang et al. [140] with permission from *Food Chemistry*, copyright 2024). (**B**) Biochemical pathways influencing core microbial reactions and vinegar quality during fermentation (reproduced from Shi et al. [130] with permission from LWT, copyright 2022).

Nutrients present in raw materials are converted into unique flavor-creating components (volatile or nonvolatile) of vinegar such as alcohols, aldehydes, phenols, organic acids, reducing sugars, esters and amino acids (Figure 2A). Hence, flavor quality and sensory perception of vinegars are directly affected by microbial composition and quantity [140]. Moreover, understanding the specific microbial genes, proteins, enzyme systems, and pathways during the vinegar fermentation helps to select and modify the excellent prominent microorganisms, which in turn positively affect the final quality of vinegar. For instance, lengthy start-up times can be reduced by controlling the ethanol oxidation pathway enzyme and cofactor Pyrroloquinoline quinine (PQQ) of *Acetobacter*, and alleviating conflict among the increase of acetic acid production and cell fitness reduction [130]. Figure 2B depicts the biochemical pathways that affect the reaction mechanisms of the main microorganisms during vinegar fermentation. The types of vinegars and methods employed for quality production are summarized in Table 3.

### 4.2. Vinegar Safety Performance Improvement

Vinegars have side effects alongside their beneficial health effects. The amount of consumption, concentration, duration, age, pregnancy case, etc. are determining factors for consumer selection. In particular, health side effects like bowel movements and increased frequency of burping or flatulence were reported for apple vinegar. This effect was observed with the consumption of larger doses of apple cider (>4 tablespoons daily) [146]. Undiluted or the consumption of more than two tablespoons daily might cause episodes of hypoglycemia in patients with insulin-dependent diabetes [147]. However, other studies conducted on animals have reported that there is no adverse effects caused by the consumption of 14 mL apple vinegar/kg body weight daily for 18 weeks (equivalent to 1120 mL apple vinegar daily for a person with 80 kg weight) [148]. Hlebowicz et al. [149] studied the effect of apple cider vinegar on rats with delayed gastric emptying for diabetes mellitus patients. They reported that patients with insulin-dependent diabetes mellitus and diabetic gastroparesis are significantly affected by delayed gastric emptying, and thus face problems with glycemic control.

Many quality and safety issues of vinegars could be produced by undesirable microorganisms during fermentation and/or aging/maturation. Vinegar quality and safety issues triggered by microbial contaminations (caused by *Bacillus* spp., *Acetobacter* spp., and *Lactobacillus* spp.) include swollen vinegar, stickiness, gas-producing contaminations (*Acetobacter* spp., *Bacillus* spp. and *Lactobacillus* spp.), and turbidity [150]. These vinegar spoilages caused by *Lactobacillaceae* lower the vinegar total sugar and furfural, while a gas-producing bacterium known as *Acetilactobacillus jinshanensis* subsp. *aerogenes* can arise throughout the vinegar fermentation process. Although *Acetilactobacillus jinshanensis* subsp. *aerogenes* is acid resistant, complete deactivation through heating (60 °C) is effective.

The safety of vinegar can be improved through technology-based microbial strain selection, proper maturation/aging and subsequent aging by adding oak chips. In particular, Lalou et al. [151] produced persimmon balsamic vinegar through pretreatment with oak chips, which accelerated the aging conditions.

The enrichment of the health-boosting and safety properties of vinegar’s bioactive components via ultrasound treatment has been achieved [152]. In this study, ultrasound treatment was applied for the enrichment of the bioactive components of tomato vinegar, which then showed positive health effects. Moreover, Yıkmış et al. [153] investigated the ultrasound treatment of verjuice vinegar commonly produced from unripe grapefruit juice. The Verjuice vinegar produced by ultrasound treatment enriched its bioactive components which showed anticarcinogenic effects.

## 5. Vinegar Health Benefits

The principal functional goods of vinegars include antimicrobial, antioxidant, blood glucose and lipid metabolism control, weight loss, anti-inflammatory and anticancer potential.

### 5.1. Anti-Microbial Activity

Yagnik et al. [154] investigated the impact of apple cider vinegar (ACV) on the growth of two resistant bacterial strains— methicillin-resistant *S. aureus* (MRSA) and *E. coli* resistant to cefepime-enmetazobactam and cefepime. The proteome approach of MRSA and *E. coli* displayed the inactivation of key metabolic enzymes for respiratory proteins and the replication of DNA. ACV perforated microbial cell membranes and organelles and therefore could alter vital proteins expression. This led to a reduction in the expression of proteins and the identification of only the ribosomal subunit proteins in *E. coli*. In MRSA, extension factor phosphoglycerate kinase OS and iNOS present the sole existing proteins. Jia et al. [155] demonstrated that *Eucommia ulmoides* leaves in vinegar incurred an antibacterial impact via a destructive effect on the bacterial cell wall and cell membrane, increasing cell permeability and leading to structural lesions and the release of cell mechanisms, thus causing cell death.

Grain vinegars can efficiently inhibit respiratory pathogens viz. *M. catarrhalis*, *S. albus*, *D. pneumonia*, and *A. streptococcus*; however, apple vinegar powerfully inhibits the growth of pathogenic bacteria like *S*. *epidermidis*, *P*. *aeruginosa*, *P. mirabilis*, and *K. pneumoniae*. Al-Rousan et al. [55] demonstrated that vinegar at 0.4% can decrease *S. typhimurium* and *E. coli* O157:H7 development and can prevent *Yersinia enterocolitica* from growing at 0.156% (*v*/*v*) [55]. On the other hand, some studies have demonstrated that several polyphenols present in vinegar are liable for its developed antibacterial effect. Reygaert and Jusufi [156] have reported that epicatechin-3-gallate may prevent microbial species at a concentration of 720 µg/mL. In addition, epigallocatechin-3-gallate can inhibit *S. aureus*, *S. mutans*, *E. coli* O157:H7, and *P*. *aeruginosa* at 0.1, 0.1, 0.5, and 0.5 μg/mL, respectively [157]. In addition to these phenolic compounds, aldehydes and alcohols present in vinegar also play a key role in its antibacterial effects. Aldehydes can damage microbial DNA and interfere with cellular processes, while alcohols disrupt cell membranes, leading to cellular leakage and inhibition of microbial growth. These combined actions enhance the overall antimicrobial efficacy of vinegar [158,159].

Vinegar’s antifungal potential has been documented. For instance, Yagnik et al. [160] reported an MIC for ACV equal to 250 µg/mL against *C. albicans*. In another study, wood vinegar derived from cocoa pod shells presented Φ inhibitory zones covering up to 12 mm against *C. albicans*, and 14 mm against *A. niger*, which corresponds to 10% [161]. Additionally, in the investigation of Oramahi and Yoshimura [162], the 1% wood *Vitex pubescens vahl* vinegar was capable of preventing *Fomitopsis palustris.* Chien et al. [163] have pointed out that wood bamboo vinegar might stop *Trichoderma viride* development. According to Shiah et al. [164], organic acids and phenolics intervene with fungal wall cells, consequently augmenting their permeability. In vinegars, organic acids can prevent bacterial development via four paths [56]: (i) the destruction of the external bacterial membrane, (ii) the inhibition of the macromolecular synthesis, (iii) the increase of intracellular osmotic pressure, and (iv) the production of antibacterial peptides. Firstly, a reduction in the intracellular pH provokes the protonation of the phosphate and carboxyl groups of lipopolysaccharides of the bacterial cell membrane and the stability is consequently impaired [165]. Likewise, the lower intracellular pH alters the enzymatic activities and can stop DNA replication and transcription, as well as protein expression [166]. To stabilize the intracellular pH, bacteria must liberate H ions via active transport, but this route absorbs ATP, affecting the standard bacterial growth [56]. Moreover, anions detached from organic acids and K ions must be liberated from the extracellular fluid, which considerably expands the intracellular osmotic pressure and results on the cell membrane breakage [16]. Along with the direct growth inhibition, other organic acids, such as butyric acid and lactic acid, lead the host cells to generate antimicrobial peptides, which damage the bacterial external membrane and inhibit their growth at gene transcription [16].

The antibacterial activities of the polyphenols present in vinegars are principally achieved by terminating the integrity of the cell membrane and meddling with the enzymes activities present in bacteria [167]. Polyphenols associate with the peptidoglycan and phospholipid bilayer of the outer membrane of bacteria and this could reduce the cell membrane integrity [167]. By virtue of their polyol function, polyphenols disturb the bacterial intracellular enzymes by acting with the amino and carboxyl groups of proteins and the chelating transition metal ion (coenzyme) [168], inhibiting the bacteria growth. The antibacterial activities of melanoidins extant in vinegar are due to their aptitude to chelate metal ions [16]. With a negative charge, melanoidins have robust chelating aptitudes for metal ions [169]. At small concentrations, melanoidins chelate iron ions, influencing their absorption and utilization, which prevent the growth of bacteria. At high levels, melanoidins damage the cell membrane of bacteria by chelating magnesium ions, subsequently leading to bacterial death [169].

### 5.2. Antioxidant Potential

Considering the apropos antioxidant activity (Antiox. Act) outcomes of diverse fruit vinegars, Kelebek et al. [87] have stated that the grape vinegars Antiox. Act, expressed by DPPH, extended from 5 to 14 mM Trolox/L and from 7–18 mM Trolox/L (ABTS). Regarding apple vinegars, this activity corresponds to 2–15 mM Trolox/L (DPPH) and 4–20 mM Trolox/L (ABTS). In the studies conducted by Xia et al. [170], the antioxidant potential of traditional balsamic vinegar was improved with ripening time. By comparing industrial processes, it appeared that traditional vinegar showed the highest Antiox. Act compared with manufacturing vinegars [92]. On the other hand, many investigations have shown that vinegar phenolic substances possess an elevated correlation with antioxidant potential [90,91]. According to Tagliazucchi et al. [117], it has been validated that the Antiox. Act of traditional balsamic vinegar is principally linked to the melanoidin and polyphenolic fractions. Xia et al. [56] have stated that the polysaccharide fraction of buckwheat vinegar was essentially composed of the Ara, Xyl, Glu, Man, and Gal and presented a good antioxidant power. By examining the in vitro digestion impact of the digestion on the jujube vinegar antioxidant activity, Li et al. [54] confirmed that, while gastric digestion decreased the content of total polyphenolics (TPC) at 55%, gastric acid and gastric protease could preserve a high antioxidant level (DPPH). In this study, during intestinal digestion, TPC was augmented by ~9%, and DPPH· was also enhanced to 24% in the first 30 min. Furthermore, cell culture and animal research established that polyphenols in fruit and grain vinegars may decrease the free radical damage [171,172] and protect the hepatocytes versus free radical damage through the nuclear factor erythroid-2-related factor 2 (Nrf2) signal route [90].

### 5.3. Anti-Inflammatory Activity

Several studies have shown that fruit vinegar could govern the proinflammatory cytokine product [173,174], enhance intestinal permeability and govern the microbiota, thus hindering the access of damaging elements of the bloodstream and monitoring the inflammation progression, as reported by Meng et al. [175]. Furthermore, it should be noted that fruit vinegar consumption frequently decreases the inflammatory cytokines, cyclooxygenase (COX)-2, nitric oxide (NO), inducible nitric oxide synthase (iNOS), and mitogen-activated protein kinases (MAPKs) [176,177]. The acetic acid in vinegars controls the blood glucose levels by the following pathways [16]: (1) hindering gastric emptying, (2) preventing disaccharidase action, (3) enhancing insulin sensitivity, and (4) supporting the glycogen production.

Throughout the vinegar aging progression, vinegar covers remarkable amounts of functional and living microorganisms and lipopolysaccharides [178]. By promoting the phagocytic impact and supporting the immune system, these molecules regulate macrophage function and control allergy, cancer, and inflammation [154]. As an illustration, nipa vinegar influences gut microbiota, expanding the population of *Proteobacteria phylum Verrucomicrobia*, and diminishing the gut *Firmicutes/Bacteroidetes* rate [171]. Some studies have revealed that the organic acids present in vinegars control the gut community by governing the gastrointestinal pH and increasing pancreatic activity [179]. Furthermore, organic acids prevent the occupation and adherence of invasive and pathogenic microorganisms [180], and boost barrier function and intestinal morphology [181]. As a model, the research reported by Jiang et al. [182] demonstrated that vinegar enhanced the detachment of lamina propia of the intestinal mucosa and the inflammatory cell permeation in the reduction of wall intestinal: p65 and ICAM-1 expression, and augmented E-cadherin in the rats’ intestinal tract [182]. Additionally, the control of the intestinal microbiota recovered the course of short-chain fatty acid (SCFA) production concerned with the mechanism of the inflammatory pathways that engaged in several diseases [183].

Likewise, vinegar improves diverse diseases in which the inflammatory action arranges their development, including arthritis, atopic dermatitis, colitis, and oxidative stress [184]. Fruit vinegar was considered to govern the production of inflammatory markers, increase the immune reaction, and ensure the gut microbiota that play an essential part in asthma pathophysiology [185]. During 24 days, oak wood vinegar reduced the IgE production in a 2,4-dinitrochlorobenzene (DNCB)-induced contact dermatitis mice model [186]. Moreover, nipa vinegar effectively showed its capability to overturn the expression of inflammatory intermediaries like iNOS and NF-kB, inducing the NO levels to decrease. In this line, NO at high levels circuitously triggers Th2 cells, which are involved in asthma physiopathology [187].

To reduce the inflammatory cell permeation and arthritic index in rats [188], the capacity of a pioneering preparation of a Pangolin-scale-processed (PSP) vinegar was investigated. PSP dropped the serum levels of inflammatory cytokines, comprising TNF-α and IL-1ß [188]. 

**Figure 3:**
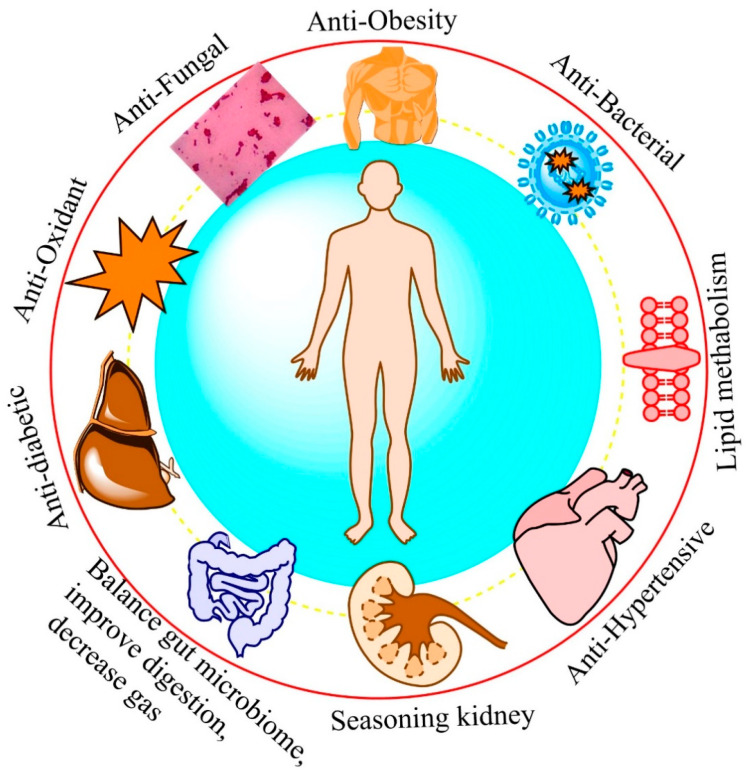
Functional qualities and health benefits of vinegar for human consumption.

In an animal model, tetramethylpyrazine, an active compound of vinegar, was associated with the inhibition of acute pancreatitis by hindering nuclear factor-kappa B (NF-ĸß) [189]. Choi et al. [176] noticed that vinegar amended pro-inflammatory markers viz. NO, iNOS, TNF-α IL-6, and MCP-1, which are involved in the inflammatory reaction. Furthermore, ligustrazine has displayed promise with regard to acute pancreatitis, promoting acinar cell apoptosis at the initial step and attenuating the Erk MAP and p38 pathways [190]. Table 4 summarizes some examples of vinegar functions on health. Figure 3 represents the functional qualities and health benefits of vinegar for human consumption.

## 6. Conclusions

This review highlights vinegar’s remarkable versatility and potential health benefits driven by its bioactive components, including organic acids, polyphenols, melanoidins, and tetramethylpyrazine. These compounds collectively contribute to vinegar’s antimicrobial, antioxidant, and anti-inflammatory properties, as well as its potential roles in glucose regulation, lipid metabolism, and weight management. LABs, crucial to the fermentation process, contribute not only to vinegar’s unique sensory qualities but also to its functional benefits. Additionally, LAB’s presence enhances the probiotic potential of vinegar, while melanoidins and polyphenols contribute significantly to its antioxidant capacity. The antibacterial and antioxidant health effects of vinegar are mostly due to the presence of organic acids, polyphenols and melanoidins. The antioxidant ability of vinegars are mainly derived from polyphenols and melanoidins, which are affected by the raw materials and fermentation status, respectively. During fermentation, the properties of some vinegars on lipid metabolism regulation, blood glucose control, and weight loss are due to the existence of acetic acid, mainly synthesized by AAB. Though these findings were predominantly derived from animal or cell trials, an elucidation of the relevant fundamental ingredient remains indistinct. Because of its characteristics, compounds and mechanisms, vinegar is not just an acidic flavoring but could also be developed as a functional food. By means of the discovery of various functional ingredients and the clarification of their mechanisms, some vinegars and their derivatives could be employed as pharmacological and therapeutic agents to prevent chronic diseases such as cardiovascular disease and diabetes.

Vinegar production methods, such as traditional aging and modern fermentation optimization, influence its bioactive profile and health benefits. Advances in fermentation technology, including microbial selection, controlled fermentation environments, and novel maturation techniques, are essential for ensuring quality and safety while enhancing vinegar’s health-promoting properties. These research pathways will aid in the development of thought on the ecological purposes and application potential of LAB, providing new insights and technologies for the fields of food industry and biotechnology.

Overall, this review underscores the diverse applications of vinegar and supports its inclusion in diets aimed at health improvement. Further research is encouraged to explore vinegar’s therapeutic applications in clinical settings and its potential as a functional supplement in food and nutrition.

## Figures and Tables

**Table 1:** Organic acids in different types of vinegars.

Vinegars	Organic Acids	References
Traditional balsamic vinegar	Citric, tartaric, gluconic, malic, succinic, lactic and acetic, formic, and tartaric acids	[70,71,72]
Alcohol vinegar	Acetic acid	[73]
Cider vinegar	Acetic, citric, formic, lactic, malic, and succinic acids	[74]
Wine vinegar	Tartaric, malic, lactic, acetic, citric, succinic and formic acids	[64,74]
Tomato vinegar	Acetic, citric, formic, lactic, malic, and succinic acids	[74,75]
Plum vinegar	Acetic, tartaric, and lactic acids	[76]
Balsamic vinegar	Acetic, formic, citric, lactic, malic and succinic, tartaric, propanoic acid, 2 methylpropanoic acid, butanoic acid, 3-methylbutanoic acid, (E)-but-2-enoic acid, hexanoic acid, octanoic acid, 4-oxopentanoic acid, furan-2-carboxylic acid and 2-phenylacetic acids	[67,71,77]
Persimmon vinegar	Acetic, lactic, quinic, tartaric, propanedioic, malic, and succinic acids	[65]
Malt vinegar	Acetic, citric, lactic, and succinic acids	[73]
Apple vinegar	Acetic, lactic, quinic, tartaric, propanedioic, malic, succinic and citric acids	[65]
Kiwifruit vinegar	Acetic, lactic, quinic, tartaric, propanedioic, malic, succinic and citric acids	[65]
Sherry vinegar	Acetic, tartaric, lactic, malic, and citric acids	[78]
Zhenjiang vinegar	Acetic, 2-methyl propionate, 3-methylbutanoic, caproic, octanoic and propionic acids	[63]
Shanxi aged vinegar	Acetic, propionic, butyric acid, 3-methyl butyric, pentanoic, hexanoic, lactic, succinic, tartaric and citric acids	[66]
Black wolfberry vinegar	Lactic, acetic, sorbic, ascorbic, succinic, oxalic, malic, citric, tartaric and γ-aminobutyric (GABA) acids	[61]

**Table 3:** Types of vinegar and quality production methods.

Types of Vinegars	Bacterial Strain Used for Fermentation	Methods Employed	Quality Improvement Applied	References
Black vinegar	*Acetobacter pasteurianus*	Saccharification of rice by *Aspergillus oryzae*	A year of aging	[141]
Cereal vinegar	*Acetobacter* sp.	Submerged fermentation of rice wine (*Oryza sativa* L.)		[142]
Balsamic vinegar	*Gluconacetobacter europaeus* and/or *Acetobacter malorum*	Spontaneous acetification of cooked grape using seed vinegar	Aged in wood barrels	[143]
White and red wine vinegar	*Gluconacetobacter europaeus* and/or *Acetobacter malorum*	Surface culture or submerged culture acetification	Aged in wood barrels (oak, chestnut, acacia and cherry)	[144]
Mature vinegar (Zhenjiang vinegar maturation)	*Acetobacter*, *Lactobacillus*, *Gluconoacetate*, *Bacillus*	Surface culture or submerged culture acetification	Aging by ultrasonic treatment	[63,145]
Aromatic vinegar (Zhenjiang aromatic vinegar)	*Lactobacillus acetotolerans*, *Lactobacillus plantarum*, *Lactobacillus reuteri*, *Lactobacillus fermentum*, *Acetobacter pasteurianus*	Solid-state fermentation	Addition of ground herbs	[29]
Fruit vinegar	Wild acetic bacteria strains	Acetification of pineapple pulp and peel wines using wild acetic bacteria strains	Leaf extract of Red-Jambo, *Syzygium malaccense*	[139]

**Table 4:** Health benefits of vinegar.

Vegetable Vinegars	Target sActivity	Objective of the Study	Main Findings	References
Leaves of *Eucommia ulmoides*	Antibacterial potential	Study the mechanism of action against *B. subtilis*	↗ Antibacterial effect and yeast Cell wall and cell membrane were damaged ↗ Cell permeability	[155]
Grape, apple, artichoke, pomegranate, apple lemon, hawthorne and sour cherry	Antimicrobial potential and antiradical activity	Distinguish between the traditional and industrailised Turkish vinegars.	↗ Antimicrobial activity in traditional vinegars compared with industrial ones ↗ Antiradical potential of pomegranate vinegars compared with industrial vinegars	[11]
*Black vinegar*	Antiox. Act and in vivo lipid lowering	Investigate these activities via a hamster model	↘ Weight gain ↗ Lipid contents and hepatic Antiox. Act	[191]
Tomato	Lipid and Glu metabolic enzyme	-Mechanism investigation -Arbitrated the anti-insulin and anti-obesity effects	↘ Fat accumulation Variations arbitrated by PPARa and AMPK increased expression	[192]
Tomato	Anti-obesity impact	Assess the efficiency in lipid accumulation	Inhibition of lipid formation	[193]
Cereal	Hepatoprotective impact	Explore the hepatoprotective impact	Changes in gut microbiota	[194]
Cereal	Impact on the spontaneous colitis impact	Exploration of the process of spontaneous colitis and investigate the variations in the epithelial wall function, inflammation and gut microbiota	Improvement of epithelium damage, Inhibition of myeloperoxidase activity and malondialdehyde (MDA)	[195]
Shanxi-aged vinegar	Anti-inflammatory activities	Investigation of the inflammatory mechanism	Enhancement of the lipid, inflammatory stress and oxidative stress.	[196]
Shanxi-aged vinegar	Impact on gut microbiome and metabolome	Investigation of the immune/inflammation factors and exploration of the in vivo impact of vinegar on gut microbiome and metabolome	↘ Inflammatory factors Vinegar consumption changed gut microbiota structure	[197]
Orange, mango, cherry and banana	Antioxidant potential	Investigation of the kinetics, chemical profile and Antiox. Act	Total antioxidant activity was assessed at 8 and 40 times greater than a commercial vinegar.	[13]
Orange	Antioxidant potential	Investigation of the fluctuations of chemical profile and Antiox. Act during fermentation	Antiox. Act was linked to ascorbic acid and phenolic compounds levels.	[198]
Rosehip fruit (*Rosa canina* L.)	Antioxidant potential	Expose the chemical profile and Antiox. Act	↗ Antioxidant activity linked to the ↘ of flavonoids	[199]
black tea	Antioxidant potential	-Evaluation of the chemical profile and Antiox. Act	↗ Organic acid contents, volatile compounds and the antioxidant activity	[200]
Apple cider	Antioxidant potential	Investigation of the change of antioxidant properties and bioactive compounds	→Antioxidant activity and phenolic substances during the acetic acid fermentation.	[201]
green jujube	Antioxidant potential	Study the impact of the in vitro gastrointestinal digestion on the Antiox. Act and hypolipidemic potential	↗ Correlation between Antiox. Act, TPC, TFC, and total acid contents Weak correlation with cholesterol adsorption capacity/antioxidant capacity	[54]
nipa palm	Antioxidant and anti-tyrosinase activities	-Characterization of chemical profile, Antiox. Act, and anti-tyrosinase potential. -Performance of molecular docking study and molecular dynamic simulation	Concentration-dependent anti-tyrosinase activity and antioxidant potential	[202]
Wood	Antimicrobial and anti-inflammatory potential	-Asses the in vivo inflammatory activity of the mammalian macrophages and antimicrobial activity against pathogenic bacteria and fungi	Stimulation of mammalian macrophages by lipopolysaccharide High antimicrobial activity but no anti-bacteriophage activity	[203]
*Cudrania tricuspidata* fruits (CTFVs)	Anti-Inflammatory potential	In vitro anti-inflammatory impacts	CTFV reduced inflammatory reaction by improving inflammatory factors	[176]
vinegar-baked Radix Bupleuri (VBCP)	Anti-Inflammatory potential	-Study the impact of extraction techniques on the physicochemical properties and biological activities of VBCP	Ammonia-assisted extraction is an effective tool to achieve high anti-inflammatory	[204]
Apple	Antioxidant, antimicrobial, antidepressant and anti-inflammatory activities	Study the biological activity of different apple cultivars, as well as physicochemical attributes and chemical composition	↗ Antidepressant impact Effective against bacteria ↗ Antioxidant activity	[205]
Curcuma phaeocaulis	Anti-angiogenic effect	Evaluation of anti-angiogenic impact and toxicity of C. phaeocaulis via zebrafish and rat models.	↘ Toxicity and ↗ anti-angiogenic activity	[206]

↗ = Increase, ↘ = Decrease, → = Steady.

## Data Availability

No new data were created or analyzed in this study. Data sharing is not applicable to this article.
